# Systemic challenge with the TLR3 agonist poly I:C induces amplified IFNα/β and IL-1β responses in the diseased brain and exacerbates chronic neurodegeneration

**DOI:** 10.1016/j.bbi.2010.04.004

**Published:** 2010-08

**Authors:** Robert Field, Suzanne Campion, Colleen Warren, Carol Murray, Colm Cunningham

**Affiliations:** a School of Biochemistry and Immunology, Trinity College Institute of Neuroscience, Trinity College Dublin, Dublin 2, Ireland; b Weatherall Institute of Molecular Medicine, University of Oxford. Oxford, OX3 9DS, UK

**Keywords:** Dementia, Delirium, Prion, Alzheimer’s disease, Infection, Inflammation, Type I interferon, Cytokine, Microglia, Priming

## Abstract

The role of inflammation in the progression of neurodegenerative disease remains unclear. We have shown that systemic bacterial insults accelerate disease progression in animals and in patients with Alzheimer’s disease. Disease exacerbation is associated with exaggerated CNS inflammatory responses to systemic inflammation mediated by microglia that become ‘primed’ by the underlying neurodegeneration. The impact of systemic viral insults on existing neurodegenerative disease has not been investigated. Polyinosinic:polycytidylic acid (poly I:C) is a toll-like receptor-3 (TLR3) agonist and induces type I interferons, thus mimicking inflammatory responses to systemic viral infection. In the current study we hypothesized that systemic challenge with poly I:C, during chronic neurodegenerative disease, would amplify CNS inflammation and exacerbate disease. Using the ME7 model of prion disease and systemic challenge with poly I:C (12 mg/kg i.p.) we have shown an amplified expression of IFN-α and β and of the pro-inflammatory genes IL-1β and IL-6. Similarly amplified expression of specific IFN-dependent genes confirmed that type I IFNs were secreted and active in the brain and this appeared to have anti-inflammatory consequences. However, prion-diseased animals were susceptible to heightened acute sickness behaviour and acute neurological impairments in response to poly I:C and this treatment also accelerated disease progression in diseased animals without effect in normal animals. Increased apoptosis coupled with double-stranded RNA-dependent protein kinase (PKR) and Fas transcription suggested activation of interferon-dependent, pro-apoptotic pathways in the brain of ME7 + poly I:C animals. That systemic poly I:C accelerates neurodegeneration has implications for the control of systemic viral infection during chronic neurodegeneration and indicates that type I interferon responses in the brain merit further study.

## 1 Introduction

Despite a huge number of published papers on inflammatory processes during chronic neurodegeneration in the last 20 years, it remains unclear how inflammation contributes to progression of neurodegeneration (Wyss-Coray, 2006). We have used the ME7 model of murine prion disease to demonstrate that microglia, the major macrophage population of the brain, are primed by ongoing neurodegeneration and amyloidosis to produce exaggerated responses to systemic challenge with the bacterial endotoxin, lipopolysaccharide (LPS). In this context the term microglial priming, derived from the widely used term macrophage priming, signifies a markedly increased ability of microglia from ME7 animals to express interleukin-1β (IL-1β) in response to LPS when neither ME7 nor LPS alone are sufficient to effect IL-1β synthesis (Cunningham et al., 2005a). This further stimulation of primed microglia results in acute neuronal death and accelerated progression of disease (Cunningham et al., 2009). Based on those ME7 studies we have since shown that either acute or chronic systemic inflammation is associated with more rapid cognitive decline in Alzheimer’s disease patients (Holmes et al., 2003, 2009). Similarly it is well known that delirium, commonly triggered by systemic infection in the demented population, accelerates progression of AD (Fong et al., 2009). Thus, further studies of the mechanisms by which systemic inflammation exacerbates underlying CNS pathology may yield insights into the role of inflammation in progression of chronic neurodegeneration in CNS disease.

Exacerbation of chronic CNS pathology by systemic gram-negative bacterial stimulation is not specific to the ME7 model of prion disease: this been replicated in many animal models of chronic neurodegeneration including Parkinson’s disease, Amyotrophic lateral sclerosis, AD and ageing (Sly et al., 2001; Nguyen et al., 2004; Godbout et al., 2005; Kitazawa et al., 2005; Godoy et al., 2008). There is also evidence that infection with neurotropic viruses such as herpes simplex virus-1 and cytomegalovirus can exacerbate cognitive decline (Strandberg et al., 2003), but surprisingly, given their high frequency in the aged and demented population, systemic viral infections have been relatively overlooked. The synthetic double-stranded RNA (dsRNA), poly inosinic:polycytidylic acid (poly I:C), potently induces the type I interferons α and β and other inflammatory cytokines (Jacobs and Langland, 1996; Matsumoto and Seya, 2008) and has been used to mimic the acute phase of viral infection but its effects on the brain remain little investigated (Katafuchi et al., 2003; Traynor et al., 2006; Cunningham et al., 2007; Konat et al., 2009). TLR3 stimulation induces a much more robust anti-viral response than TLR4 stimulation (Doyle et al., 2003) and this is characterised by high expression of type I interferons.

In the current study, we hypothesized that the neurodegenerating brain is primed with respect to stimulation by systemic anti-viral mimetics. Thus, we predicted that ME7 prion-diseased animals would show similar systemic cytokine responses but amplified CNS inflammatory and sickness behavioural responses to systemic poly I:C stimulation, with respect to normal animals given the same stimulus. We have examined the CNS inflammatory profile and in particular, have focussed on type I interferons and downstream pathways. We also predicted that poly I:C would accelerate disease progression but have no lasting consequences for normal animals.

## 2 Materials and methods

### 2.1 Animals and stereotaxic surgery

Female C57BL/6 mice (Harlan, Bicester, UK), were housed in groups of five and given access to food and water *ad libitum*. We used females in order to avoid fighting and injury, which has significant effects on behaviour. Animals were kept in a temperature-controlled room (21 °C) with a 12:12 h light–dark cycle. The mice were anaesthetised intraperitoneally (i.p.) with Avertin (2,2,2-tribromoethanol) and positioned in a stereotaxic frame. Two small holes were drilled in the skull either side of the midline to allow for bilateral injection of 1 μl of a 10% w/v ME7-infected C57BL/6 brain homogenate made in sterile PBS. Injections were made into the dorsal hippocampus (co-ordinates from bregma: anteroposterior, – 2.0 mm; lateral, – 1.6 mm; depth, – 1.7 mm) using a microsyringe (Hamilton, Reno, Nevada) with a 26 gauge needle. Control animals were injected with a 10% w/v normal brain homogenate (NBH) in PBS, derived from a naive C57BL/6 mouse. All procedures were performed in accordance with United Kingdom Home Office and Republic of Ireland Department of Health & Children licenses and all efforts were made to minimise both the suffering and number of animals used.

### 2.2 Systemic poly I:C challenges

Poly I:C was obtained from Amersham Biosciences (Little Chalfont, Buckinghamshire, UK). It was prepared for injection by resuspending in sterile saline, heating to 50 °C at a concentration of 2 mg/ml to ensure complete solubilisation and then allowing to cool naturally to room temperature to ensure proper annealing of double-stranded RNA. Poly I:C was stored at −20 °C until use. Experimental groups at 18 weeks post-inoculation with ME7 or NBH were challenged intraperitoneally (i.p.) with either poly I:C (12 mg/kg) or sterile saline to examine systemic and CNS inflammatory responses to systemic poly I:C. All animals for which mRNA data was analysed were challenged only once with poly I:C (at 18 weeks). Dose response studies have previously been performed (Cunningham et al., 2007) and this dose produces hypothermia and weight loss in normal mice that is comparable with that induced by infection with influenza virus at 0.1 of its LD_50_ (McKinstry et al., 2009). The choice of 18 weeks post-ME7 inoculation as the point for systemic challenge for mRNA transcriptional analysis was based on our previous finding that robust priming of microglia occurs at this time (Cunningham et al., 2005a). Animals were initially perfused at 6 h post-poly I:C to capture the time point at which qualitative and quantitative differences were apparent in the hypothermic response (from preliminary data) and subsequently, further animals were perfused at 4 h to examine earlier gene expression. In a subset of animals repeated systemic challenges were made at 14, 16 and 18 weeks to examine the effect of repeated “viral stimulation” on the progression of neurodegenerative disease. In these animals, temperature responses and acute neurological deficits were measured after each of the three challenges. The data have been presented for temperature at 14 weeks and neurological changes at 16 weeks since these were the earliest time points in disease at which robust changes were evident. The two weeks interim period allowed full recovery from each systemic inflammatory response before initiation of subsequent challenges. Tissue for analysis of histological changes was taken at 3 or 15 h post-poly I:C to examine microglial and inflammatory markers and neurodegenerative changes, respectively.

### 2.3 ELISA for cytokines

Under terminal anaesthesia the thoracic cavity was opened and blood collected into heparinised tubes directly from the right atrium of the heart. This procedure was carried out 6 h post-poly I:C challenge. Whole blood was centrifuged to remove cells and the remaining plasma aliquoted and stored at −20 °C before assay. These samples were then analysed for IL-6, TNF-α and IFN-β (IL-1β levels were previously determined to be considerably lower after poly I:C challenge). Samples were serially diluted to verify linear responses and were quantified only if the absorbance fell on the linear portion of the standard curve. The IFNβ assay kit was supplied by Biosource (Nivelles, Belgium) and mouse IL-6, and TNF-α were measured using R&D systems “duo-set” kits. Protocols followed for these assays were as previously published (Cunningham et al., 2007). The reliable quantitation limit of all assays was 15.6 pg/ml.

### 2.4 Immunohistochemistry for IBA-1, COX-2, IL-1β, IRF3, caspase-3, TUNEL

ME7 and NBH animals were deeply anaesthetised and transcardially perfused with heparinised saline followed by 10% formal-saline at 18 weeks post-inoculation and at 3, 6 or 15 h post-poly I:C or saline for histology experiments. Tissue was paraffin wax-embedded and 10 μm coronal sections of fixed tissue at the level of the hippocampus were cut on a microtome, dewaxed in xylene and rehydrated through alcohols of decreasing concentration. Primary antibodies were obtained from Santa Cruz (Santa Cruz, CA, US: COX-2), Millipore (activated caspase-3: Temecula, CA, USA), Peprotech (London, UK: IL-1β), Abcam (Cambridge UK: IBA-1), Invitrogen (NY, USA: IRF3), Promega (TUNEL, Southampton, UK). Biotinylated secondary antibodies, normal sera, and avidin–biotin complex were from Vector Laboratories (Peterborough, UK). Avidin-horseradish peroxidase was obtained from DAKO (Cambridge, UK). Immunohistochemistry for all antigens was carried out by the Avidin–Biotin-Complex (ABC) method with minor modifications, depending on the antibody used, and has been described in detail in previous publications (Cunningham et al., 2005a). Cell counting was performed for IL-1b, TUNEL and IBA-1. For IL-1b and TUNEL, positive cells were identified and counted throughout the hippocampus and thalamus of all animal groups. For IBA-1, a 0.62 × 0.47 mm section of the centre of the dorsal hippocampus, (containing the CA1 pyramidal layer and stratum oriens and radiatum, but not the dentate gyrus granule layer) was photographed and used for microglial counts in NBH and ME7 animals treated with poly I:C. Positively stained cells were identified and counted using the analyse particles function in Image J software (rsbweb.nih.gov).

### 2.5 RNA extraction and Taqman reverse transcription-PCR

Animals challenged intraperitoneally with poly I:C (12 mg/kg) or saline were terminally anaesthetised at 4 and 6 h after poly I:C and then transcardially perfused with heparinised saline. Brains were rapidly removed, hippocampi and hypothalami dissected out, placed in eppendorf tubes, snap frozen on liquid nitrogen and stored at −80 °C until further use.

Total RNA was extracted from brain samples using Qiagen RNeasy® Plus mini kits (Qiagen, Crawley, UK) according to the manufacturer’s instructions. Contaminating genomic DNA was eliminated via degradation during extraction using the Qiagen RNase-free *DNase1* enzyme. Approximate yields were determined by spectrophotometry at 260 and 280 nm. RNA was stored at −80 °C until cDNA synthesis and PCR assay.

All equipment and reagents were supplied by Applied Biosystems (Warrington, UK) unless otherwise stated. Assays for IFN-α, IFN-β, IL-10, IP-10, IRF-7, TLR3, RIG-I, PKR, OAS, Mx1, Bax, Fas, IFNγ, and all T cell transcripts were designed using the published sequences for these genes, applied to Primes Express™ software. Where possible, probes were designed to cross an intron such that they were cDNA specific. All primer pairs were checked for specificity by standard reverse transcription (RT)-PCR followed by gel electrophoresis. Each primer pair produced a discrete band of expected amplicon size. We subsequently learned that C57 mice have a non-functional Mx1 protein due to a deletion in exons 9 though 11 in the *Mx1* gene (Staeheli and Sutcliffe, 1988; Jin et al., 1998). Our primers for this gene were designed such that they are specific to an unaffected region of the gene and span the boundary of exons 2 and 3. For Taqman PCR, cDNA was generated from total RNA using a High Capacity cDNA Reverse Transcription Kit (Applied Biosystems). Two hundred nanograms of total RNA were reverse transcribed in a 10 μl reaction volume. One microliter of the RT reaction (equivalent to 20 ng of RNA) was subsequently used for the PCR, as described previously (Cunningham et al., 2007). The housekeeping gene glyceraldehyde-3-phosphate dehydrogenase (GAPDH) was measured in each sample using Applied Biosystems Rodent GAPDH Taqman Kit. All PCR data were normalised to the expression of GAPDH. More detailed description of these methods, and full primer sequences, are available in supplemental information.

### 2.6 Body temperature

Core body temperature was measured using a thermocouple rectal probe (Thermalert TH5, Physitemp, Clifton, New Jersey). Temperature measurements were made on three separate occasions in the week prior to poly I:C injections to habituate mice to the procedure and thus minimise the effects of stress. Temperatures were recorded at baseline and then at 4, 9, 13 and 24 h following i.p. challenge with poly I:C.

### 2.7 Motor co-ordination: horizontal bar and inverted screen

Following systemic challenge with poly I:C, ME7 or NBH-inoculated mice were assessed for their co-ordination of motor function. The horizontal bar was designed to assess forelimb muscle strength and co-ordination, and consisted of a 26 cm long metal bar, 0.2 cm diameter, supported by a 19.5 cm high wooden column at each end. Each mouse was held by the tail, placed with its front paws at the central point of the bar, and rapidly released. Mice were scored based on whether they fell, held on for 60 s, or reached a platform on a supporting column, with the latter two results scoring the maximum of 60 s. The inverted screen (Kondziela, 1964) assessed muscular strength for all four limbs. It consisted of a wooden frame, 43 cm square, covered with wire mesh (12 mm squares of 1 mm diameter wire). The mouse was placed on the screen and this was then slowly inverted. The time it took for the mouse to fall was measured, up to a criterion of 60 s. Padding was provided to cushion mice falling from either apparatus.

### 2.8 Statistics

Behavioural data was analysed by repeated measures ANOVA with Bonferroni post hoc analysis after significant main effects. Peripheral ELISA data and CNS transcription data were analysed by two-way ANOVA with ME7/NBH and poly I:C/saline or time post-poly I:C as factors, with Bonferroni post hoc tests. One-way ANOVAs were also performed where the inclusion of multiple time points post-poly I:C did not allow a full factorial analysis. Cell counts were analysed by one-way ANOVA for IBA-1, IL-1β and TUNEL.

## 3 Results

### 3.1 Anti-viral response in the hippocampus and hypothalamus

**Fig. 1:**
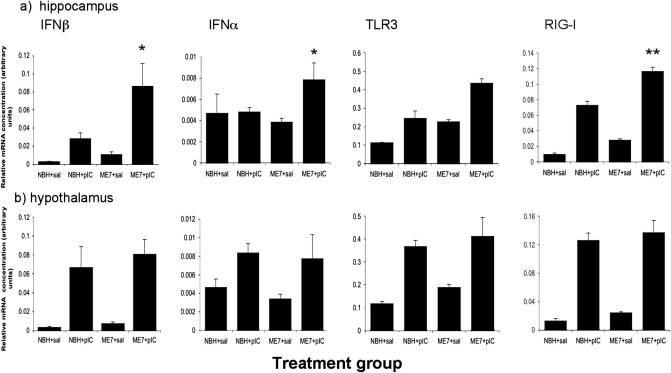
Systemic poly I:C induces anti-viral transcripts in the brain. Expression of mRNA for IFNβ, IFNα, TLR3 and RIG-I in the hippocampus (a) or hypothalamus (b) of NBH and ME7 animals (6 hours post-challenge, 18 weeks post-inoculation) treated once with saline or poly I:C (12 mg/kg). Data are expressed as mean ± SEM, *n* = 3 for NBH + saline and *n* = 5 for all other groups. Significant interaction of poly I:C and ME7 in the hippocampus by two-way ANOVA is indicated by ^∗^*p* < 0.05 and ^∗∗^*p* < 0.01. Interactions between factors were not observed in the hypothalamus and main effects of poly I:C are described in the text.

Intra-peritoneal treatment of NBH and ME7 animals (18 weeks post-inoculation) with poly I:C resulted in the robust transcription of IFNβ in the hippocampus 6 h following administration of poly I:C (Fig. 1a). IFNβ was transcribed more robustly in ME7 animals than in NBH animals given the same poly I:C challenge. There was an effect of disease (*F* = 7.93, df 1, 14, *p* = 0.0137), an effect of poly I:C (*F* = 17.82, df 1, 14, *p* = 0.0009) and an interaction of these two factors (*F* = 4.68, df 1, 14, *p* < 0.05) by two-way ANOVA for IFNβ. The hippocampal induction of IFN-α was less marked and more variable. Nonetheless, there was an interaction between disease and poly I:C for this gene (*F* = 5.68, df 1, 14, *p* < 0.05). TLR3 mRNA was induced in the hippocampus both by poly I:C treatment and by ME7. Two-way ANOVA revealed a main effect of both poly I:C (*F* = 41.38, df 1, 14, *p* < 0.0001) and of ME7 (*F* = 24.3, df 1, 14 *p* = 0.0002) but there was no significant interaction, although TLR3 was induced further by poly I:C challenge in ME7 animals (one-way ANOVA, ME7 + poly I:C versus NBH + poly I:C *p* < 0.01 and versus ME7 + saline *p* < 0.001). RIG-I showed similar expression to IFNβ, with main effects of disease (*F* = 59.21, df 1, 14, *p* < 0.0001) and of poly I:C (*F* = 351.86, df 1, 14, *p* < 0.0001) and a significant interaction of these two factors (*F* = 9.97, df 1, 14, *p* < 0.01). Thus anti-viral responses were amplified in ME7 + poly I:C animals with respect to NBH + poly I:C.

These transcripts (IFNβ, IFNα, TLR3, RIG-I) were also examined in the hypothalamus since this region is highly sensitive to circulating inflammatory mediators. Poly I:C induced robust transcription of all 4 genes in the hypothalamus, but this transcription was equivalent in ME7 and NBH animals. These data are shown in Fig. 1b. Two-way ANOVAs for these genes showed that there were main effects of poly I:C in all cases, but no effect of ME7 and no interaction between the two factors (*F* = 1.62, df 1, 14, *p* > 0.22 in all cases). Thus, the exaggerated anti-viral response of ME7 animals, to poly I:C, is present in the hippocampus, but not in the hypothalamus.

### 3.2 Systemic cytokines

The levels of IFNβ, TNF-α and IL-6 were elevated in the plasma of poly I:C-treated animals (6 h post-treatment) but were below detectable levels in both NBH and ME7 animals challenged with sterile saline (Table 1). Poly I:C groups were significantly different to relevant saline controls for IFNβ (*p* < 0.001), TNF-α (*p* < 0.01) and IL-6 (*p* < 0.05) by Bonferroni post hoc tests. Treatment with poly I:C did not produce significantly different cytokine levels in NBH versus ME7 animals (*p* > 0.05 for all three cytokines). Therefore, systemic cytokine responses to poly I:C are not significantly different in animals with prior neurodegeneration.

### 3.3 Core-body temperature response to poly I:C

**Fig. 2:**
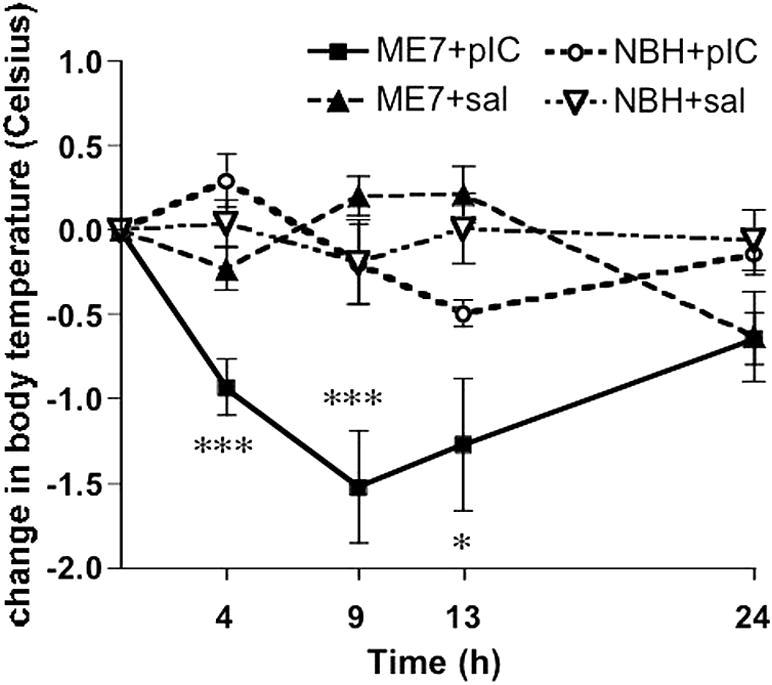
Poly I:C induces marked hypothermia in ME7 animals. Animals were treated once with poly I:C (12 mg/kg) at 14 weeks post-inoculation with ME7 or NBH. Core-body temperature was taken by rectal probe at 0, 4, 9, 13 and 24 h post-poly I:C or saline. *n* = 10 for all groups except NBH + poly I:C (*n* = 14). Data are expressed as mean ± SEM. Significant differences between ME7 + poly I:C and corresponding NBH + poly I:C time point by Bonferroni post hoc test, after significant two-way ANOVA, are denoted by ^∗∗∗^*p* < 0.001, ^∗∗^*p *< 0.01, ^∗^*p *< 0.05. Full ANOVA analyses are in the main text.

At the earliest time point examined (14 weeks post-inoculation with ME7, 4 h after poly I:C), poly I:C induced the predicted mild hyperthermic response in normal (NBH) animals but caused hypothermia in prion-diseased (ME7) animals. In addition, the later hypothermic phase was exaggerated in ME7 animals with respect to NBH animals treated with poly I:C (Fig. 2). Repeated measures ANOVA revealed a significant effect of time (*F* = 5.66, df 4, 160, *p* < 0.0005), a significant effect of treatment (*F* = 9.29, df 3, 40, *p* < 0.0001) and an interaction of treatment and time (*F* = 6.46, df 12, 160, *p* < 0.0001). Bonferroni post hoc tests revealed that ME7 + poly I:C animals were significantly different to NBH + poly I:C animals at 4 h, 9 h (*p* < 0.001) and 13 h (*p* < 0.05) and significantly different to ME7 + saline animals at 9 and 13 h (*p* < 0.001). Conversely ME7 + saline were not different to NBH + saline at any time point (*p* > 0.05). Similar early and exaggerated hypothermic responses were seen after poly I:C challenge to ME7 animals at 16 and 18 weeks (data not shown).

### 3.4 CNS inflammatory cytokine responses to systemic poly I:C

**Fig. 3:**
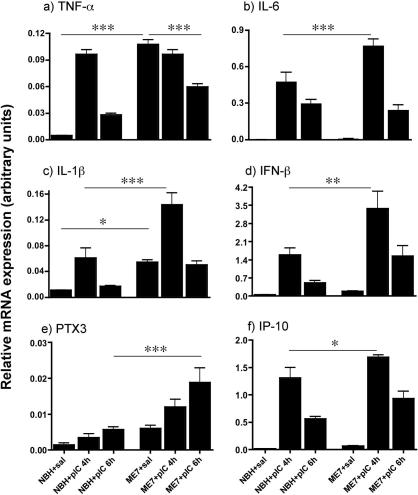
Inflammatory cytokine and NFκB- and IRF3-dependent gene transcription. Hippocampal expression of mRNA for TNF-α (a), IL-6 (b), IL-1β (c) IFNβ (d), PTX3 (e) and IP-10 (f) at 4 and 6 h post-challenge of NBH and ME7 animals (18 weeks post-inoculation) with poly I:C. *n* = 5 for all 6 h animals, *n* = 4 for both 4 h groups, *n* = 5 for ME7 + saline and *n* = 3 for NBH + saline. CNS cytokine mRNA levels were measured by quantitative PCR from ME7 and NBH animals. Significant differences, by Bonferroni post hoc test after significant one-way ANOVA, between ME7 + poly I:C and NBH + poly I:C time point are denoted by ^∗∗∗^*p *< 0.001, ^∗∗^*p *< 0.01, ^∗^*p *< 0.05. Full ANOVA analyses are in the main text.

As shown in Fig. 3 poly I:C induced differential hippocampal responses in NBH and ME7 animals 18 weeks post-inoculation. TNF-α mRNA was markedly induced in ME7 animals *per se* (Fig. 3a). One-way ANOVA (*F* = 51.85, df 5, 26, *p* = 0.0001) with selected Bonferroni post hoc tests revealed that ME7 + saline was significantly different to NBH + saline. Systemic challenge with poly I:C induces opposite effects on TNF-α in NBH and ME7 animals. Levels in ME7 + poly I:C animals were actually depressed at 4 h with respect to ME7 animals and statistically significantly lower at 6 h (*p* < 0.001 by one-way ANOVA with Bonferroni post hoc test).

Poly I:C induced very marked increases by 4 h in IL-6 in the hippocampus of both NBH and ME7 animals (Fig. 3c). The increase was, however, more marked in ME7 + poly I:C animals. A significant one-way ANOVA (*F* = 65.01, df 5, 26, *p* < 0.0001) with selected Bonferroni pairwise tests revealed no difference between IL-6 levels in NBH + saline and ME7 + saline animals (*p* > 0.05), but showed that ME7 + poly I:C at 4 h was significantly different to NBH + poly I:C (*p* < 0.001) and these levels decreased somewhat by 6 h.

IL-1β mRNA was clearly induced in the hippocampus of ME7 animals at 4 h post-poly I:C and returned to near baseline levels by 6 h in normal animals. The poly-I:C-induced increase was markedly higher in ME7 animals (Fig. 3b). One-way ANOVA (*F* = 24.54, df 5, 26, *p* < 0.0001) followed by selected Bonferroni post hoc comparisons showed that ME7 + saline was significantly higher than NBH + saline (*p* < 0.05). The IL-1β increase post-poly I:C was more marked in ME7 than in NBH (*p* < 0.001).

IFNβ, which is IRF3-dependent, was induced more markedly in the hippocampus of ME7 animals treated with poly I:C (Fig. 3d) and appeared to peak at 4 h. A significant one-way ANOVA (*F* = 18.45, df 5, 25; *p* < 0.0001) followed by Bonferroni post hoc tests revealed that ME7 + poly I:C was significantly higher than NBH + poly I:C at their peak values (*p* < 0.01), but ME7 + saline and NBH + saline were not significantly different (*p* > 0.05).

PTX3, an NFκB-dependent gene with no reported regulation by IRF3, showed an exaggerated induction in the hippocampus of ME7 + poly I:C compared to NBH + poly I:C. Levels of this transcript were still rising at 6 h (Fig. 3e), distinct from the NFκB-dependent, primary response genes IL-1β, TNFα and IL-6 (Fig. 2a and b) and consistent with secondary induction by IL-1β. Selected Bonferroni post hoc comparisons after a significant one-way ANOVA (*F* = 9.27, df 5, 25, *p* < 0.0001) revealed that ME7 + saline was not significantly different to NBH + saline but that ME7 + poly I:C was significantly different to NBH + poly I:C at 6 h (*p* < 0.001).

We also examined the time course of hippocampal expression of the NFκB and IRF3-dependent gene interferon-inducible protein 10 (IP-10). This chemokine mRNA showed a very similar temporal pattern of induction to the other primary response genes studied (Fig. 3f), peaking at 4 h and decreasing thereafter, making it unlikely that it is induced by IFNβ. After a significant one-way ANOVA (*F* = 67.76, df 5, 25, *p* < 0.0001), Bonferroni post hoc tests showed that ME7 + poly I:C was significantly higher than NBH + poly I:C but ME7 + saline was not significantly different to NBH + poly I:C (*p* > 0.05).

### 3.5 Histological analysis of cellular changes

**Fig. 4:**
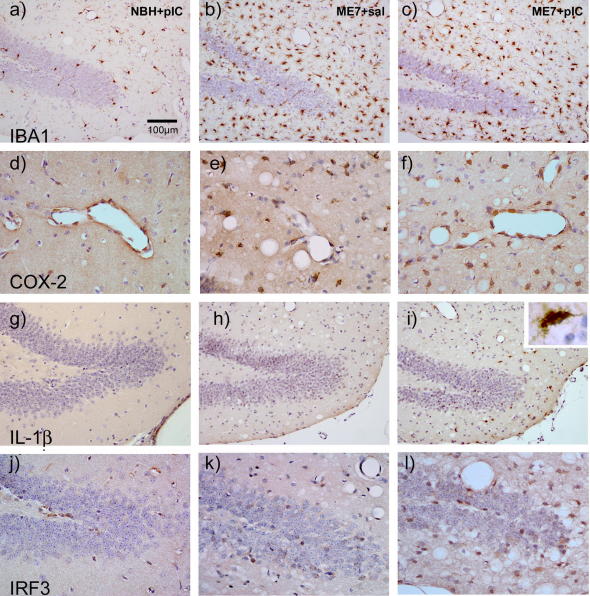
Microglia are primed with respect to responses to systemic poly I:C. Microglial priming and microglial/endothelial activation was assessed 3 h after poly I:C or saline in NBH and ME7 animals. (a–c) IBA-1 labelling of microglial cells in NBH + poly I:C, ME7 + saline and ME7 + poly I:C animal groups, showing more condensed morphology and increased numbers in ME7 with respect to NBH. (d–f) COX-2 labelling shows few microglia in NBH animals but activated endothelium after poly I:C. Conversely, ME7 + saline shows increased microglia but absent endothelial staining, while ME7 + poly I:C animals show both populations are COX-2-positive. (g–i) IL-1β labelling is absent in both NBH + poly I:C and ME7 + saline animals but ME7 + poly I:C (i) shows positive cells of microglial morphology (inset) in periventricular and perivascular areas and around the dentate gyrus. (j–l) IRF3 labelling in scattered cells in NBH + poly I:C and in ME7 + saline and in greater numbers in periventricular areas and around the dentate gyrus in ME7 + poly I:C animals. Scale bar = 100 μm in (a–c) and (g–i) and 50 μm in (d–f) and (j–l).

IBA-1, COX-2 and IL-1β staining illustrated clear morphological evidence of microglial activation (Fig. 4 a versus b and c) and increased expression of COX-2 (d and e) but an absence of IL-1β-positive cells (g and h) in ME7 animals with respect to NBH controls 3 h after treatment with saline or poly I:C. IBA-1 revealed significantly increased numbers of activated microglia (*p* < 0.001 ANOVA with Bonferroni post hoc test; Table 2) in ME7 animals compared to NBH with no further increase following administration of poly I:C (p≫0.05). Upon systemic challenge with poly I:C these microglial cells, in the periventricular and dentate gyrus regions, now synthesised detectable levels of IL-1β (i) in ME7 but not NBH animals. IL-1β positive cells were found to be significantly higher in number in ME7 animals challenged with poly I:C than all other groups (*p* < 0.05 by ANOVA with Bonferroni post hoc test; Table 2). The endothelial cell layer was also induced to synthesize COX-2 in response to systemic poly I:C in both NBH and ME7 animals (d and f). Quantification of individual COX-2-labelled cells is not straightforward in the tightly apposed endothelial layer of hippocampal vessels, but it is clear that the vast majority of hippocampal vessels are positively labelled after poly I:C challenge in NBH and ME7, while those in the ME7 + saline group are not. Numerous cells in periventricular and perivascular areas and around the dentate gyrus showed IRF3 labelling, and there was evidence of more intense and more frequent staining of nuclei in the hippocampus and thalamus, consistent with nuclear translocation in the areas of prior ME7-associated pathology. There were no gross changes in the hippocampal levels of PrP^Sc^ in response to systemic poly I:C challenge (Supplementary data).

### 3.6 Evidence for exaggerated IFNβ production and action in the CNS

**Fig. 5:**
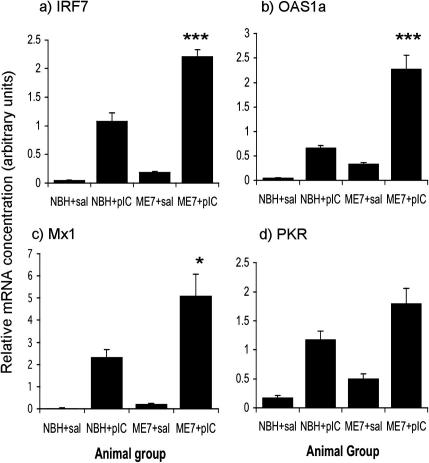
IFNβ-responsive, stat 1/2-dependent, gene transcription. Expression of mRNA for the IFNβ-responsive genes IRF7 (a), OAS1a (b), Mx1 (c) and PKR (d) was examined in the hippocampus 6 h post-challenge with poly I:C or saline in ME7 and NBH animals 18 weeks post-inoculation. Data are expressed as mean ± SEM, *n* = 3 for NBH + saline and *n* = 5 for all other groups. Significant interactions between disease (ME7) and poly I:C by two-way ANOVA are indicated by ^∗^*p *< 0.05 and ^∗∗∗^*p *< 0.0005.

Fig. 5(a–d) shows evidence of increased IFNα/β action in the hippocampus via expression of IRF7, OAS, PKR and Mx1 transcription. These genes are known to be IFNβ-responsive, STAT1/2-dependent genes and are not induced directly by TLR3 signalling or by IRF3 activation (Honda and Taniguchi, 2006). IRF7 was clearly induced by poly I:C (main effect of poly I:C: *F* = 231.16, df 1, 14, *p* < 0.0001). There was also a main effect of disease (*F* = 39.84, df 1, 14, *p* < 0.0001) and an interaction of disease and poly I:C (*F* = 23.98, df 1, 14, *p* < 0.0005). Similarly OAS1a showed much higher induction in ME7 + poly I:C animals than in NBH + poly I:C animals. There were main effects of disease (*F* = 43.96, df 1, 14, *p* < 0.0001) and of poly I:C (*F* = 79.41, df 1, 14, *p* < 0.0001) and an interaction of these two factors (*F* = 21.32, df 1, 14, *p* < 0.0005). Likewise, Mx1, assessed at the exon 2–exon 3 junction, showed an exaggerated induction in ME7 animals treated with poly I:C. There were main effects of disease (*F* = 7.70, df 1, 14, *p* < 0.05) and of poly I:C (*F* = 45.29, df 1, 14, *p* < 0.0001) and an interaction of these two factors (*F* = 5.87, df 1, 14, *p* < 0.05). Finally, PKR was more robustly induced by poly I:C in ME7 animals than in NBH animals. There were main effects of disease (*F* = 9.51, df 1, 14, *p* < 0.01) and of poly I:C (*F* = 55.12, df 1, 14, *p* < 0.0001), but no significant interaction (*F* = 0.89, df 1, 14, *p* = 0.36) in this case. Thus, there is exaggerated type I IFN action in the CNS of ME7 animals challenged with poly I:C with respect to NBH animals similarly challenged.

### 3.7 Evidence of anti-inflammatory activation: additional IFNβ effects?

**Fig. 6:**
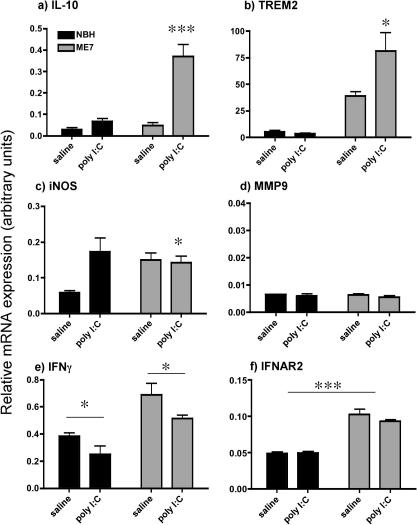
Evidence of anti-inflammatory profile post-IFNβ induction. Expression of mRNA for (a) IL-10, (b) TREM2, (c) iNOS, (d) MMP9, (e) IFNγ and (f) IFNAR2 as assessed by quantitative PCR at 6 h after a single challenge with poly I:C (12 mg/kg) or saline in ME7 and NBH animals at 18 weeks post-inoculation. Data were analysed by two-way ANOVA and significant interactions between disease and poly I:C are in panels (a and b) and (c). Main effects of poly I:C are shown in (e) and (a) main effect of disease is shown in (f), as assessed by Bonferroni post hoc test. The degree of statistical significance is illustrated by ^∗^*p *< 0.05 and ^∗∗∗^*p *< 0.001, respectively.

IL-10 was modestly induced by both poly I:C in normal animals (*F* = 34.97, df 1, 12, *p* < 0.0001) and by disease (main effect of disease: *F* = 28.32, df 1, 12, *p* = 0.0002) (Fig. 6a). There was also an interaction of disease and poly I:C, ME7 + poly I:C showing considerably more marked induction than all other groups (*F* = 22.23, df 1, 12, *p* = 0.0005).

TREM2 (Fig. 6b) was markedly induced by disease (two-way ANOVA main effect of disease (*F* = 34.13, df 1, 12, *p* = 0.0001), and was slightly, but not significantly, affected by poly I:C (*F* = 4.49, df 1, 12, *p* = 0.0576). However there was a significant interaction between disease and poly I:C. TREM2 was markedly more elevated in ME7 + poly I:C than in any other group (*F* = 5.32, df 1, 12, *p* = 0.0415).

The expression of iNOS was increased by poly I:C in NBH animals but was not increased by poly I:C in ME7 animals (Fig. 6c). As such, there were no main effects of disease or poly I:C but an interaction between these (*F* = 5.22, df 1, 14, *p* = 0.0385).

The expression of MMP9 was very low and was not altered by any treatment (Fig. 6d). There were no statistically significant changes.

IFNγ (Fig. 6e) was modestly increased in ME7 animals (main effect of disease, *F* = 21.34, df 1, 14, *p* = 0.0004) and decreased by poly I:C (main effect of poly I:C: *F* = 6.3, df 1, 14, *p* = 0.025). There was no interaction between these factors.

Thus, in addition to reduced TNF-α expression (Fig. 3), there are further anti-inflammatory changes that appear to be selectively apparent in ME7 animals upon poly I:C treatment. Heightened expression of the signalling type I interferon receptor, IFNAR2 in ME7 animals (Fig. 6f) may contribute to this. IFNAR2 was induced by prion disease (main effect of disease: *F* = 107.98, df 1, 12, *p* < 0.0001) but is not significantly affected by poly I:C (*F* = 0.79, df 1, 12, *p* = 0.39).

### 3.8 Neurological consequences

**Fig. 7:**
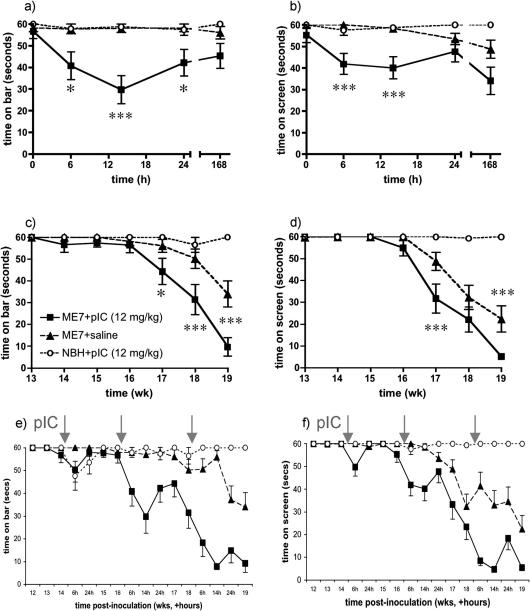
Poly I:C induces acute neurological impairment in ME7 animals and accelerates disease progression. (a and b) ME7 and NBH animals were challenged i.p. with poly I:C (12 mg/kg) at 16 weeks and their performance on the horizontal bar (a) and inverted screen (b) tests of motor co-ordination and muscle strength was assessed at 6, 14, 24 and 168 h post-challenge. (c and d) The same tests were conducted weekly to assess the influence on disease progression of poly I:C or saline challenges at 14, 16 and 18 weeks post-inoculation with ME7 or NBH. Longitudinal studies (c and d) experiments were conducted with *n* = 12 NBH + pIC and *n* = 15 for all other groups. Data are shown as mean ± SEM and statistically significant differences by Bonferroni post hoc test after significant main effects and interactions by repeated measures ANOVA are indicated by ^∗^*p *< 0.05, ^∗∗∗^*p *< 0.001. (e and f) These data are also shown with both acute and weekly time point assessments to depict the course of neurological impairments when underlying disease and systemic challenge combine. Treatment with poly I:C is indicated by grey arrows and in longitudinal experiments animals were treated three times with poly I:C.

Despite these anti-inflammatory effects, presumably resulting from IFNα/β action, animals challenged with poly I:C showed acute and chronic exacerbations of disease. ME7 and NBH animals were challenged with poly I:C (12 mg/kg) or saline at 14, 16 and 18 weeks post-inoculation with ME7 or NBH were assessed for performance on muscle strength and motor co-ordination tasks (inverted screen and horizontal bar), which are known to deteriorate with progression of the ME7 strain of prion disease but to be intact at 16 weeks (Betmouni et al., 1999; Cunningham et al., 2005b). Poly I:C significantly impaired performance of ME7 animals on both the inverted screen and horizontal bar at 16 weeks post-inoculation (Fig. 7a and b). Neither co-ordination nor muscle strength were acutely affected in poly I:C-treated NBH animals or in ME7 + saline animals. Repeated measures ANOVA analysis of acute effects on the horizontal bar revealed main effects of treatment (*F* = 11.86, df 2, 38, *p* < 0.0001) and of time (*F* = 3.34, df 4, 156, *p* < 0.05) and an interaction of these two factors (*F* = 3.03, df 8, 156, *p* < 0.005). Bonferroni post hoc tests showed that ME7 + poly I:C animals were significantly impaired with respect to both other groups at 6 h (*p* < 0.05), 14 h (*p* < 0.001) and 24 h (*p* < 0.05). Similarly, on the inverted screen there were significant main effects of time (*F* = 5.04, df 4, 156, *p* < 0.001), of treatment (*F* = 13.19, df 2, 38, *p* < 0.0001) and an interaction of treatment and time (*F* = 2.58, df 8, 156, *p* < 0.05). Bonferroni post hoc tests showed that ME7 + poly I:C animals were significantly impaired compared to ME7 + saline at 6 and 14 h (*p* < 0.001) and were impaired compared to NBH + poly I:C at 6 h (*p* < 0.05) and 14 h (*p* < 0.01).

Despite these acute impairments most animals recover their baseline performance at 1 week post-challenge (168 h). However, longitudinal analysis of performance on bar and screen tasks showed that repeated challenge with poly I:C (at 14, 16 and 18 weeks) resulted in more rapid development of permanent loss of function on these tasks. Repeated measures analysis of weekly performance in the same animals revealed clearly exacerbated neurological decline as measured by both tasks (Fig. 7c and d). There were main effects of treatment (*F* = 17.12, df 2, 38, *p* < 0.0001) and of time (*F* = 30.05, df 7, 266, *p* < 0.0001) and an interaction of these factors (*F* = 9.25, df 14, 266, *p* < 0.0001) on bar performance. Bonferroni post hoc tests revealed significant differences between ME7 + poly I:C and ME7 + saline from 17 weeks onwards (*p* < 0.05 at 17 weeks and *p* < 0.001 from 18 weeks). Similar analysis of inverted screen data revealed main effects of treatment (*F* = 30.35, df 2, 38, *p* < 0.0001), of time (*F* = 61.72, df 7, 266, *p* < 0.0001) and a significant interaction (*F* = 16.27, df 14, 266, *p* < 0.0001). Bonferroni post hoc tests showed significant differences between ME7 + poly I:C and ME7 + saline at 17 and 19 weeks (*p* < 0.001).

Thus poly I:C induced acute, neurological impairments and also exacerbated progression of disease in ME7 animals, without effect in normal animals. These data have also been illustrated as repeated acute events superimposed upon longitudinal decline (Fig. 7e and f) to illustrate the influence of repeated anti-viral responses on disease course. We have demonstrated that the primary response to systemic poly I:C (i.e. peripheral induction of IFNβ) was not significantly different after one, two or three systemic challenges with poly I:C (12 mg/kg i.p.). These data are shown in Supplementary data (S3).

### 3.9 Apoptosis

**Fig. 8:**
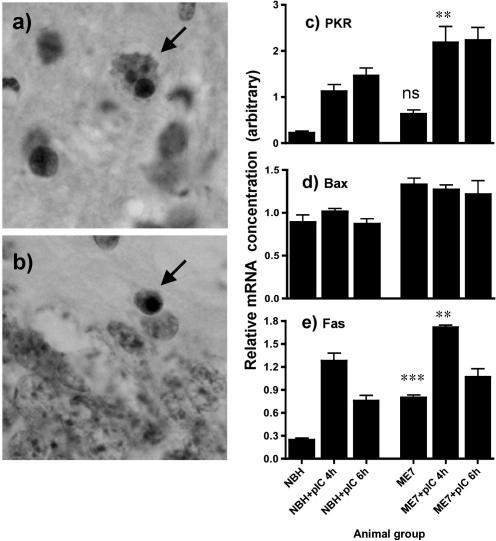
Systemic poly I:C activates pro-apoptotic pathways and increases apoptosis in ME7 animals. Examples of immunohistochemically labelled TUNEL and activated caspase-3-positive apoptotic cells are indicated in (a) and (b), respectively by arrows. (c–e) Analysis of mRNA expression of PKR, Bax and Fas at different times post-poly I:C challenge (12 mg/kg i.p.). Data were analysed by one-way ANOVA with selected pairwise Bonferroni post hoc tests. Significant post hoc tests are indicated by ^∗∗^*p *< 0.01, and ^∗∗∗^*p *< 0.001 and ns denotes a non-specific difference between NBH and ME7. Data are expressed as mean ± SEM, *n* = 3 for NBH, *n* = 4 for ME7 + pIC 4 h, NBH + pIC 4 h and *n* = 5 for all other groups.

We observed small numbers of activated caspase-3-positive cells and larger numbers of TUNEL-positive cells in ME7 animals 15 h after treatment with saline or poly I:C. Examples of both activated caspase-3 and TUNEL-positive cells are shown in Fig. 8 (a and b). The larger number and smaller size of TUNEL-positive cells reflects the later stage of cell-degeneration, as we have previously shown after LPS treatment of ME7 animals (Cunningham et al., 2005a,b). TUNEL-positive apoptotic cells (positive labelling plus condensed nucleus) were counted in the areas of pathology (the hippocampus and thalamus) in 10 μm sections of animals 15 h post-challenge with poly I:C or saline. ME7 + poly I:C animals had significantly higher numbers of apoptotic cells per 10 μm section than ME7 + saline (12 ± 3 versus 6 ± 1; *p* < 0.05 by one-way ANOVA with Bonferroni post hoc test). NBH + poly I:C animals showed very low number of apoptotic cells (1 ± 1 per 10 μm section). These data are also shown in Table 2.

We examined expression of pro-apoptotic genes PKR, Fas and Bax (Fig. 8c–e) and found a clear poly I:C-induced increase in PKR and Fas mRNA expression. Bax was induced somewhat in ME7 animals, but not elevated further by poly I:C treatment. Two time-points are provided to provide temporal information but post hoc comparisons have only been performed on the 4 h data. Disease and poly I:C influence PKR expression (*F* = 13.53, df 5, 20, *p* < 0.0001) and Bonferroni post hoc comparisons revealed that while NBH and ME7 were not significantly different, NBH + poly I:C was significantly lower than ME7 + poly I:C at 4 h (*p* < 0.05). Similar analysis of Bax revealed that NBH was significantly different to ME7 but that no further changes were induced by poly I:C treatment. Analysis of Fas data revealed a significant one-way ANOVA (*F* = 38.3, df 5, 20, *p* < 0.0001) and Bonferroni post hoc tests showed that NBH was significantly different to ME7 (*p* < 0.001) and that ME7 + poly I:C was significantly higher than both ME7 (*p* < 0.001) and NBH + poly I:C (*p* < 0.01). Thus there was increased apoptosis and amplified expression of pro-apoptotic genes in ME7 + poly I:C animals.

## 4 Discussion

In the current study we have demonstrated that acute systemic poly I:C stimulation, mimicking systemic viral infection, was associated with significantly amplified CNS IL-1β and IFNβ responses in diseased animals compared to normal animals. Transcription of several interferon-responsive genes demonstrated IFNα/β action in the brain and this was associated with a number of anti-inflammatory effects. However, the IFN-responsive pro-apoptotic genes PKR and Fas were also increased and were associated with increased apoptotic cell death. Repeated poly I:C challenges induced successive episodes of acute neurological deficits and caused a progressive acceleration of late stage disease signs without effect in normal animals. Thus systemic challenge with the TLR3 agonist poly I:C exacerbates existing chronic neurodegeneration.

### 4.1 TLR3 and the type I interferon response

Toll-like receptor-3 (TLR3) is a key pattern recognition receptor for dsRNA and poly I:C (Alexopoulou et al., 2001), although dsRNA can also be recognised by other sensors such as MDA5, RIG-I and PKR (Honda and Taniguchi, 2006; Kato et al., 2006). The robust induction of type I interferons α and β and other inflammatory cytokines by poly I:C (Jacobs and Langland, 1996; Matsumoto and Seya, 2008) makes this a useful tool with which to mimic acute phase anti-viral responses and to examine the consequences of these for CNS disease.

The stimulation of TLR3 initiates signal transduction via both NFκB and interferon regulatory factor 3 (IRF3) and the stimulation of both IRF3- and NFκB-dependent genes in the current study suggest TLR3 engagement. IRF3 is expressed constitutively and translocates to the nucleus where it induces transcription of the genes for IFNα/β. The periventricular activation of IL-1β and IRF3 suggests that dsRNA may even have some access to the parenchyma in these regions with underlying pathology. Systemic poly I:C has been reported to disrupt the blood brain barrier at 24 h post-challenge (Wang et al., 2004) and there is evidence that this barrier is already somewhat compromised in areas of existing prion disease pathology (Wisniewski et al., 1983; Chung et al., 1995). Although astrocytes and endothelial cells can respond to poly I:C *in vitro* (Ishikawa et al., 2004; Kraus et al., 2004; Farina et al., 2005), microglia have been shown to express TLR3, to respond to poly I:C (Melton et al., 2003; Olson and Miller, 2004) and to be dependent on TLR3 for responses to intracerebroventricularly administered poly I:C (Town et al., 2006).

The production of type I interferons results in signalling at the type I IFN receptor, inducing transcription of the gene for IRF7 as well as other key anti-viral transcripts, PKR, OAS and Mx1 (Honda and Taniguchi, 2006). The robust transcription of all of these genes observed here demonstrates that IFNα/β is produced in the CNS, at mRNA and protein levels, and is active in the brain. Levels of all of these transcripts are markedly increased by systemic challenge with poly I:C and this occurs to a much higher level in ME7 animals, despite similar systemic responses. Thus, the CNS produces exaggerated type I interferon responses to systemic poly I:C suggestive of a primed state of the degenerating brain with respect to subsequent responses to these challenges.

### 4.2 Microglial priming and exacerbation of CNS inflammation

We have previously shown that during chronic neurodegeneration, microglia are primed by disease to produce exaggerated sickness and CNS inflammatory responses to systemic stimulation with the TLR4 agonist LPS (Combrinck et al., 2002; Cunningham et al., 2005a). The term microglial priming is based on early descriptions of macrophage priming in which pretreatment with IFNγ primes macrophages to produce more robust responses to LPS (Johnson et al., 1983; Pace et al., 1983). Though a CNS priming factor has not yet been identified, evidence for similar in microglial priming effects, and exacerbation of pathology, has since been provided by researchers in many models of CNS pathology, including Parkinson’s disease (Godoy et al., 2008), prion disease (Cunningham et al., 2009), Wallerian degeneration (Palin et al., 2008) ageing (Godbout et al., 2005; Barrientos et al., 2006), ALS (Nguyen et al., 2004), AD (Sly et al., 2001; Kitazawa et al., 2005) and stroke (McColl et al., 2007). Thus systemic inflammatory events can accelerate neurodegenerative disease and we have recently shown that AD patients who suffer systemic inflammatory events, including infections, show more rapid progression of cognitive decline (Holmes et al., 2003, 2009).

The demonstration here that animals primed by neurodegeneration also mount exaggerated IL-1β and type I interferon responses to systemic challenge with poly I:C indicates that hyper-reactivity of these primed cells is not specific to LPS challenges. This finding therefore adds TLR3 activation to the list of pattern recognition receptors likely to be capable of exacerbating neurodegenerative disease. While this might have been predicted from our prior work with LPS/TLR4 (Cunningham et al., 2009), its demonstration is significant. We have made repeated challenges with poly I:C to demonstrate acute, reversible, exacerbations of neurological function whose magnitude depends on the severity of the underlying pathology, and have shown that these repeated challenges also accelerate disease in a cumulative manner. The repeated challenge strategy was made possible by the demonstration that these repeated treatments do not produce tolerance to poly I:C in behavioural (Cunningham et al., 2007) or peripheral type I interferon (Supplementary data) responses. Thus, 3 challenges do not appear to induce an inflammatory phenotype distinct from that induced by a single challenge. We also show that a single poly I:C challenge is sufficient to induce an acute increase in apoptosis (Fig. 8) and that three challenges are insufficient to produce any lasting impairment in normal animals (Fig. 7). Thus, our prediction is that the acute neurological deficits are produced by the exaggerated inflammatory response in areas with prior pathology and the long-term deterioration is due to the additive effects of individual discrete pathological insults superimposed on the progressing disease.

### 4.3 Mechanisms: pro- and anti-inflammatory effects

Here we have examined many potential inflammatory pathways that might explain this exacerbation of disease, including transcription of iNOS and matrix metalloproteinases such as MMP9, the induction of IFNγ and TNF-α and increased infiltration of cytotoxic T cells or natural killer cells. All of these pathways showed either no induction, or in the cases of IFNγ and TNF-α, a suppression of mRNA levels. The suppression of TNF-α and iNOS concomitant with increased IL-1β, IL-6, IFNα/β, IL-10 and TREM2 represents a post-priming inflammatory phenotype that is somewhat different to that described after LPS challenge (Cunningham et al., 2005a) and may reflect the anti-inflammatory influence of IFNα/β. Type I interferons principally orchestrate anti-viral responses but have typically been viewed as anti-inflammatory in the CNS: they limit leukocyte infiltration to the brain (Prinz et al., 2008) and reduce the expression of pro-inflammatory cytokines such as IL-17, IL-12 and TNF-α (Makar et al., 2008; Chen et al., 2009). In addition, loss of endogenous IFNβ exacerbates inflammation and pathology in the EAE model of multiple sclerosis (Teige et al., 2003). Notwithstanding any anti-inflammatory influence of IFNβ, IL-1β is elevated at mRNA and protein levels, only in the microglia of ME7 + poly I:C animals, and may be implicated in the exaggerated hypothermia observed as well as remaining a potential source of neurotoxicity that may contribute to the accelerated disease progression. IL-1β is known as an exacerbator of ischaemia-induced neurotoxicity (Rothwell and Luheshi, 2000) and an examination of poly I:C challenges and their consequences in IL-1 receptor type 1 and interferon receptor 1 deficient mice (IL-1R1^−/−^ and IFNAR1^−/−^) are now important priorities in the ME7 model.

### 4.4 Interferons, sickness behaviour, apoptosis and CNS disease

Despite some anti-inflammatory effects in the brain, type I interferon responses may still be deleterious. The use of IFN-α in cancer therapy, has taught us that systemic IFN levels lead to sickness behavioural responses and it has been shown that systemic injection of interferons can induce interferon-responsive genes in the hypothalamus (Wang et al., 2008). These data indicate that type I IFNs have actions in the CNS, but that these, like sickness behaviour in a general sense, are largely adaptive. However, there is some evidence that transgenic (Campbell et al., 1999), or viral encephalitis-induced (Sas et al., 2009) expression of IFN-α can produce CNS neuropathology. There remain limited studies of pathological effects of acute type I IFN responses in the brain.

However, there is strong evidence that IFNα/β is a potent pro-apoptotic stimulus and the marked type I interferon-dependent up-regulation of PKR observed here might be a key event with respect to neurodegeneration. PKR has been demonstrated in many studies to induce apoptosis (Balachandran et al., 1998, 2000) with up-regulation of Fas and Bax and activation of caspase-3 implicated. Neurodegeneration in the ME7 model of prion disease is via these pathways (Chiesa et al., 2005) and in the current study we have shown increased Fas mRNA synthesis and caspase-3/TUNEL-positive cell death at the histological level. Thus, the type I IFN-induced activation of PKR represents a strong possibility for induction of pro-apoptotic cascades that may accelerate the process of neurodegeneration. Thus, while type I interferons exert some anti-inflammatory effects in the current study, systemic viral infection and consequent CNS activation of pro-apoptotic pathways could still have deleterious consequences for those with existing CNS pathology.

Based on the hypothesis that prion diseases are viral infections, early studies attempted, and failed, to slow progression of disease by boosting type I interferon responses (Gresser and Pattison, 1968; Field et al., 1969; Worthington, 1972; Gresser et al., 1983). Indeed CNS treatment with poly I:C (Allen and Cochran, 1977) or adenoviral co-infection actually accelerated prion disease (Ehresmann and Hogan, 1986). Here we have made systemic challenges with poly I:C when microglial activation and synaptic and neuronal degeneration are well established and in so doing have effected an amplification of the CNS anti-viral response and an acceleration of disease. This raises the possibility that inflammatory cells recognise cellular dysfunction and mark these cells for destruction through similar pathways used to destroy virally-infected cells. Induction of some interferon-responsive genes during prion disease has previously been reported (Baker et al., 2004; Riemer et al., 2004; Stobart et al., 2007) and amplification of these responses, in the current study, is associated with increased apoptosis and disease progression.

## 5 Conclusion

Based on the findings presented here, systemic challenge with viral mimetics can accelerate neurodegenerative disease. Given the high frequency of viral infection in the ageing population it is important to assess the impact of systemic viral infection on chronic neurodegeneration in both animal models and in humans. The demonstration of similar disease exacerbation after real viral infection would constitute an important proof of the current hypothesis. Influenza, rhinoviruses and increasingly noroviruses show high prevalence in the elderly population (Estes et al., 2006) and murine-adapted strains of these viruses are available (Hyde et al., 2009; Majde et al., 2010). That systemic inflammation, triggered by diverse etiologies, can accelerate the progression of AD (Holmes et al., 2009) suggests that interventions targeting these systemic exacerbations offer opportunities to slow disease progression.

## Conflict of interest

The authors declare no conflicts of interest.

## Figures and Tables

**Table 1:** Plasma inflammatory cytokine concentrations.

Cytokine	NBH + saline	NBH + poly I:C	ME7 + saline	ME7 + poly I:C
IFNβ	ND	2567 ± 410	ND	2737 ± 694
TNF-α	ND	132 ± 24	ND	121 ± 36
IL-6	ND	1837 ± 374	ND	1739 ± 569

Systemic cytokine levels 6 h post-poly I:C or sterile saline (pg/ml). ND signifies cytokines below detection limit (not detectable).

**Table 2:** Immunohistochemical quantification.

IHC label	NBH + poly I:C	ME7 + saline	ME7 + poly I:C
IBA-1	48 ± 5	172 ± 15∗	172 ± 14∗
IL-1β	ND	ND	49 ± 29∗
TUNEL	1 ± 1	6 ± 1∗	12 ± 3∗

IL-1β and TUNEL counts were performed in the hippocampus and thalamus in 10 μm coronal sections, 3 and 15 h, respectively after poly I:C administration. IBA-1 counts were performed in a 0.62 × 0.47 mm region of the hippocampus. ND signifies that positive cells were not detectable. Significant differences to NBH + poly I:C controls by one-way ANOVA and Bonferroni post hoc tests are signified by ∗*p *< 0.05.
