# Supplementary material for: Systemic challenge with the TLR3 agonist poly I:C induces amplified IFNα/β and IL-1β responses in the diseased brain and exacerbates chronic neurodegeneration
Source: Brain Behav Immun. 2010 Aug;24(6):996–1007. doi: 10.1016/j.bbi.2010.04.004 (PMC3334265; doi:10.1016/j.bbi.2010.04.004)
Supplement: Supplementary data [file mmc1.doc]

### Supplementary material

*Quantitative RT-PCR, detailed protocol*

Animals challenged intraperitoneally with saline or poly I:C (12 mg/kg) were terminally anaesthetized at 0, 4, or 6 hours post-challenge and then transcardially perfused with heparinised saline. Brains were rapidly removed and hippocampi punched out, placed in eppendorf tubes, snap frozen on liquid nitrogen and stored at -80°C until further use. Total RNA was extracted from brain samples using Qiagen RNeasy® Plus mini kits (Qiagen, Crawley, UK) according to the manufacturer’s instructions. Contaminating genomic DNA was eliminated via degradation during extraction using the Qiagen RNase-free *DNase1* enzyme. Yields were determined by spectrophotometry at 260 nm. RNA was stored at –80°C until cDNA synthesis and PCR assay. All equipment and reagents were supplied by Applied Biosystems (Warrington, UK). Primer and probe sequences for IL-1β, IL-6, TNF-α, IFNβ, PTX-3, iNOS, Bax, Fas were as previously published (Cunningham et al., 2005, 2007, 2009, Palin 2008) while those for IL-1R1, IFNAR2, IRF7, TREM2, CD200R, OAS1a, Mx1, PKR, TLR3 were designed using the published sequences for these genes, applied to Primes Express™ software. Where possible, probes were designed to cross an intron such that they were cDNA specific. Table 1 lists the sequences for primers and probes for each assay previously unpublished. In some cases (noted below) the fluorescent DNA binding probe SYBR green has been used in place of a specific probe. All primer pairs were checked for specificity by standard reverse transcription (RT)-PCR followed by gel electrophoresis and each primer pair produced a discrete band of the expected amplicon size (supplementary information). For Taqman PCR, cDNA was generated from total RNA using a High Capacity cDNA Reverse Transcription Kit (Applied Biosystems). Two hundred nanograms of total RNA were reverse transcribed in a 10µl reaction volume. One microliter of the RT reaction (equivalent to 20 ng of RNA) was subsequently used for the PCR, as described previously (Cunningham et al., 2007).

A standard curve was constructed from total RNA isolated from mouse brain tissue 24 hours after intra-cerebral challenge with 2.5 μg LPS, which is known to up-regulate most target transcripts of interest in this study. This standard curve was constructed using a higher concentration of RNA in the reverse transcriptase reaction than that in the samples for analysis to ensure that the cytokine transcription of all experimental animal groups will fall within the range of the standard curve constructed. Serial 1 in 5 dilutions of the cDNA synthesized from brains of LPS-injected mice were made and a curve plotted of the Ct value (the cycle number at which the fluorescence of the product of transcribed gene crosses the threshold of detection) versus the log of the concentration (assigned an arbitrary value since the absolute concentration of cytokine transcripts is not known). Verification that this curve is a straight line confirms the efficiency of the PCR reaction across the entire concentration range and the equation of this line can then be used to calculate the relative concentrations of the experimental samples. Since the top standard has been assigned an arbitrary value, it follows that the calculated concentrations of all experimental samples will also have arbitrary values, but these values will be directly related to the equation of the line.

The housekeeping gene glyceraldehyde-3-phosphate dehydrogenase (GAPDH) was measured in each sample using Applied Biosystems Rodent GAPDH Taqman Kit. All PCR data were normalised to the expression of GAPDH.

# Table S1. Mouse Taqman primer and probe sequences

| Target | Oligonucleotide | Sequence | Amplicon size (bp) |
| --- | --- | --- | --- |
| OAS1a | Forward primer | 5’-CTTTGATGTCCTGGGTCATGT-3’ | 123 |
|  | Reverse primer | 5’-GCTCCGTGAAGCAGGTAGAG-3’ |  |
| PKR | Forward primer | 5’-CCGAAAACTGCCGGAACA-3’ | 97 |
|  | Reverse primer | 5’-CTGACTGGGAAACACCATTACTTG-3’ |  |
| Mx1 | Forward primer | 5’-GCCAGACGATGGATTCTGTGA-3’ | 149 |
|  | Reverse primer | 5’-TGAACTCTGGTCCCCAATGAC-3’ |  |
| Bax-α | Forward primer | 5’-GTTTCATCCAGGATCGAGCAG-3’ | 238 |
|  | Reverse primer | 5’-CCCCAGTTGAAGTTGCCATC-3’ |  |
| Fas | Forward primer | 5’-CTGCGATGAAGAGCATGGTTT-3’ | 208 |
|  | Reverse primer | 5’-CCATAGGCGATTTCTGGGAC-3’ |  |
| Trem2 | Forward primer | TGTGGTCAGAGGGCTGGACT | 68 |
|  | Reverse primer | CTCCGGGTCCAGTGAGGA |  |
|  | Probe | CCAAGATGCTGGGCACCAACTTCAG |  |
| IRF7 | Forward primer | CGAGGAACCCTATGCAGCAT | 108 |
|  | Reverse primer | TACATGATGGTCACATCCAGGAA |  |
|  | Probe | CCAGCTCTCACCGAGCGCAGC |  |
| TLR3 | Forward primer | 5’-CGGGCCGCCTTTTCA-3’ | 110 |
|  | Reverse primer | 5’-TAGTGTGGGTCTGGTTGATCCA-3’ |  |
|  | Probe | 5’-CAGTTTAGATATGCGCTTCAATCCGTTCGA-3’ |  |
| IFNAR2 | Forward primer | 5’-GAGAGCAGAAAAACGGACTTAAGAG-3’ | 98 |
|  | Reverse primer | 5’-TCGCAGACACCACAAGACACA-3’ |  |
|  | Probe | 5’-TGCACCGTCTCTGCCGTCGG-3’ |  |
| CD200R1 | Forward primer | AGGAGGATGAAATGCAGCCTTA | 80 |
|  | Reverse primer | TGCCTCCACCTTAGTCACAGTATC |  |
| IL-1R1 | Forward primer | 5’-GCAATATCCGGTCACACGAGTA-3’ | 117 |
|  | Reverse primer | 5’-ATCATTGATCCTGGGTCAGCTT-3’ |  |
|  | Probe | 5’-TCCTGAGCCCTCGGAATGAGACGATC-3’ |  |
| MMP9 | Forward primer | 5’-CGAACTTCGACACTGACAAGAAGT-3’ | 114 |
|  | Reverse primer | 5’-GCACGCTGGAATGATCTAAGC-3’ |  |
|  | Probe | 5’-TCTGTCCAGACCAAGGGTACAGCCTGTTC-3’ |  |
| Perforin | Forward primer | TTGGCCCATTTGGTGGTAAG | 52 |
|  | Reverse primer | AGTCTCCCCACAGATGTTCTGC |  |
| Granzyme A | Forward primer | CCTGAAGGAGGCTGTGAAAGAATC | 531 |
|  | Reverse primer | CCCTGCACAAATCATGTTTAGTCC |  |
| CD 8 | Forward primer | TCAAGACGGCCCTTTCTCAGT | 103 |
|  | Reverse primer | TCCCTGTCCCAAAGACCATCT |  |
| MHC 1 | Forward primer | AAGAGCAGTGGTTCCGAGTGA |  |
|  | Reverse primer | GGTTGTAGTAGCCGAGCAGGTT |  |

Where probe is not included, SYBR green has been used it its place

**Figure S1 Deposition of PrP^Sc^ is not significantly altered by repeated poly I:C stimulation**

**
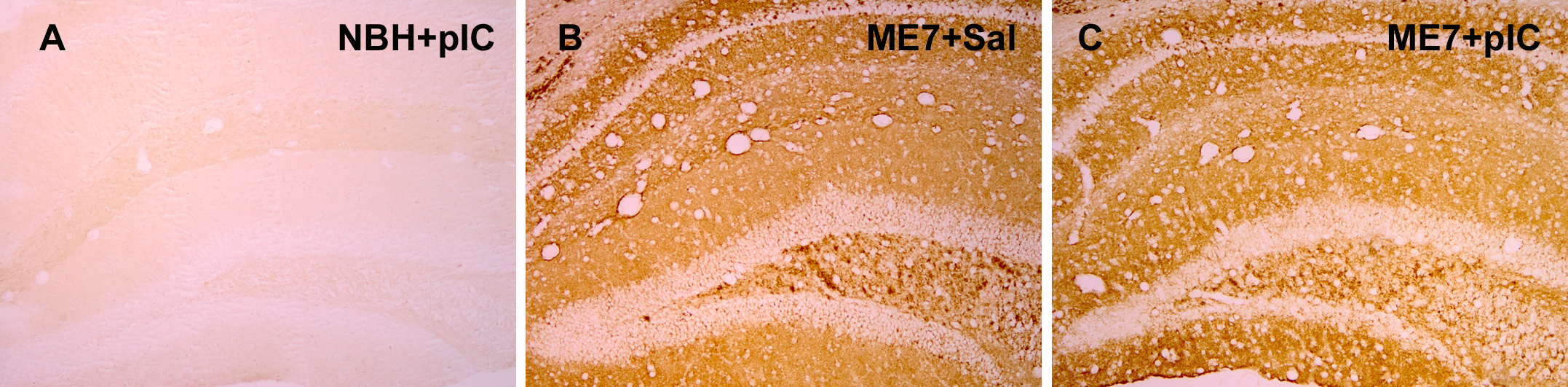
**

**Figure s3** Deposition of PrP^Sc^ is not significantly altered by repeated poly I:C stimulation. Animals were inoculated with NBH or ME7 and then treated with poly I:C or saline i.p. at 14, 16 and 18 weeks post-inoculation. Animals were sacrificed at 20 weeks and formalin fixed, wax embedded brain tissue was assessed for proteinase K-resistant prion protein (PrP^Sc^) using the 6D11 antibody. NBH tissue is devoid of PrP^Sc^ (A). ME7 animals should profound PrP^Sc^ labeling in the hippocampus and the density and distribution of this labeling is not significantly different in those ME7 animals receiving three systemic challenges with poly I:C at 12 mg/kg. Thus systemic poly I:C does not appear to influence PrP^Sc^ deposition.

Figure S2

a) CD8 b) Perforin c) MHC I

**Figure S2 mRNA expression of T cell markers.**

Gene transcription changes at 6 hours post-challenge with poly I:C or saline. n=3 for NBH+saline and n=5 for all other groups. Data were analysed by two way ANOVA with disease and poly I:C as factors. a) Disease induced CD8 expression (main effect of disease, p<0.0001, F=61.19, df 1,14) and poly I:C decreased its expression (main effect of poly IC, p<0.001, F=14.04, df 1,14) but there was no interaction between these factors. b) perforin expression was increased by disease (main effect of disease, p<0.0001, F=38.27, df 1,14) but was not affected by poly I:C and these factors did not interact. c) MHC I expression was also induced by disease (main effect of disease, p<0.0001, F=42.09, df 1,14) but was not affected by poly I:C and there was no interaction between these factors.

**Figure S3.**

**
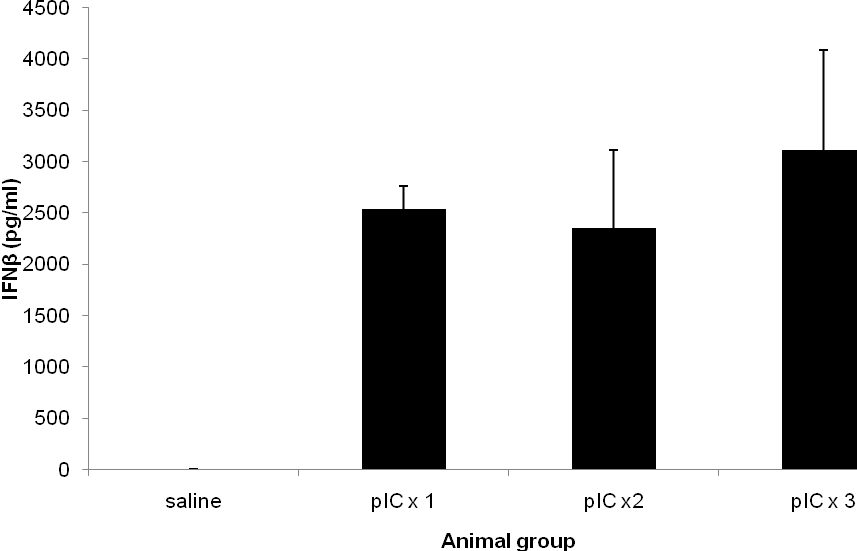
**

**Figure S3. Plasma levels of IFNβ after repeated systemic poly I:C stimulation.**

Plasma levels of IFNβ were examined following one, two or three doses of poly I:C (12 mg/kg i.p.) to determine whether repeated systemic administration induces immunological tolerance or indeed primes the peripheral immune system to produce an exaggerated systemic responses to subsequent stimulation. Each dose of poly I:C was administered two weeks apart to ensure full recovery from the prior systemic inflammatory response. One-way ANOVA comparison of plasma IFNβ levels, showed that there were no significant differences between groups challenged with one, two or three doses of poly I:C (F=0.4079, df 2,10 p >>0.05). This demonstrates that animals neither become tolerant to repeated poly I:C challenge, nor show exaggerated responses upon multiple systemic poly I:C challenges.

**Figure S4. PAGE gel for Mx1 primers.**


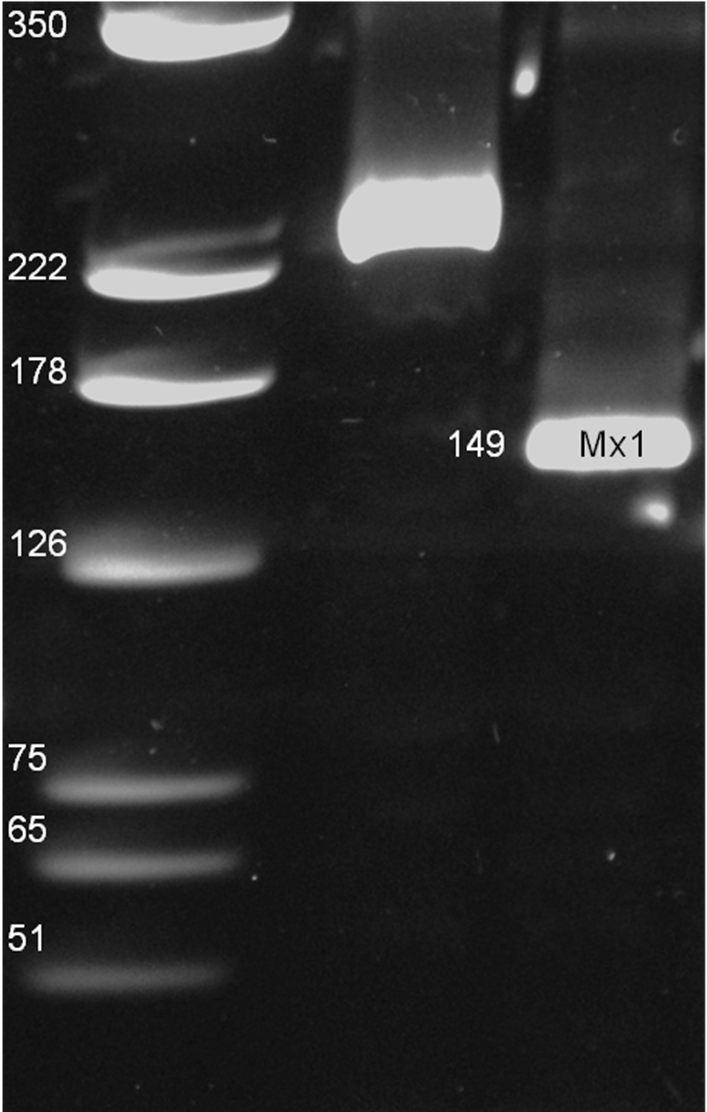
It is known that C57 mice lack exons 9 to 11 of the Mx1 gene (Staeheli, 1988) and it has been reported that Mx1 transcripts are not induced by IFN-treatment (Jin et al., 1998). However the former paper shows that IFN-α does induce transcripts of the intact portion of the gene. Our Mx1 primers were designed to cross the exon 2-exon 3 boundary within the cDNA sequence in order to be cDNA specific and these primers were checked for specificity by standard reverse transcription (RT)-PCR followed by gel electrophoresis. These primers produced a discrete band of 149 base pairs, the expected amplicon size for this gene product. This validates the use of Mx1 transcripts as a readout of type I interferon action.
